# The shared genetic basis of mood instability and psychiatric disorders: A cross‐trait genome‐wide association analysis

**DOI:** 10.1002/ajmg.b.32907

**Published:** 2022-07-15

**Authors:** Guy Hindley, Kevin S. O'Connell, Zillur Rahman, Oleksandr Frei, Shahram Bahrami, Alexey Shadrin, Margrethe C. Høegh, Weiqiu Cheng, Naz Karadag, Aihua Lin, Linn Rødevand, Chun C. Fan, Srdjan Djurovic, Trine V. Lagerberg, Anders M. Dale, Olav B. Smeland, Ole A. Andreassen

**Affiliations:** ^1^ NORMENT Centre, Institute of Clinical Medicine University of Oslo and Division of Mental Health and Addiction, Oslo University Hospital Oslo Norway; ^2^ Psychosis Studies Institute of Psychiatry, Psychology and Neurosciences, King's College London London UK; ^3^ Center for Bioinformatics, Department of Informatics University of Oslo Oslo Norway; ^4^ Department of Radiology University of California San Diego La Jolla California USA; ^5^ Multimodal Imaging Laboratory University of California San Diego La Jolla California USA; ^6^ Department of Cognitive Science University of California San Diego La Jolla California USA; ^7^ Department of Medical Genetics Oslo University Hospital Oslo Norway; ^8^ NORMENT Centre, Department of Clinical Science University of Bergen Bergen Norway; ^9^ KG Jebsen Centre for Neurodevelopmental disorders University of Oslo Oslo Norway; ^10^ Department of Psychiatry University of California San Diego La Jolla California USA; ^11^ Department of Neurosciences University of California San Diego La Jolla California USA

**Keywords:** ADHD, bipolar disorder, genetic overlap, major depression, mood instability, schizophrenia

## Abstract

Recent genome‐wide association studies of mood instability (MOOD) have found significant positive genetic correlation with major depression (DEP) and weak correlations with other psychiatric disorders. We investigated the polygenic overlap between MOOD and psychiatric disorders beyond genetic correlation to better characterize putative shared genetic determinants. GWAS summary statistics for schizophrenia (SCZ, *n* = 105,318), bipolar disorder (BIP, *n* = 413,466), DEP (*n* = 450,619), attention‐deficit hyperactivity disorder (ADHD, *n* = 53,293), and MOOD (*n* = 363,705) were analyzed using the bivariate causal mixture model and conjunctional false discovery rate methods. MOOD correlated positively with all psychiatric disorders, but with wide variation in strength (*r*_g_ = 0.10–0.62). Of 10.4 K genomic variants influencing MOOD, 4 K–9.4 K influenced psychiatric disorders. Furthermore, MOOD was jointly associated with DEP at 163 loci, SCZ at 110, BIP at 60 and ADHD at 25. Fifty‐three jointly associated loci were overlapping across two or more disorders, seven of which had discordant effect directions on psychiatric disorders. Genes mapped to loci associated with MOOD and all four disorders were enriched in a single gene‐set, “synapse organization.” The extensive polygenic overlap indicates shared molecular underpinnings across MOOD and psychiatric disorders. However, distinct patterns of genetic correlation and effect directions may relate to differences in the core clinical features of each disorder.

## 1 INTRODUCTION

Mood instability (MOOD) is a psychological construct defined as a tendency to experience frequent, rapid fluctuations of intense affect and an inability to regulate these fluctuations or their behavioral sequelae (Marwaha et al., 2014). The concept was first described in people with borderline personality disorder and is a central component of the disorder (Koenigsberg, 2010). While present in approximately 14% of the general population (Marwaha, Parsons, Flanagan, & Broome, 2013), it is also overrepresented in several other psychiatric disorders, including schizophrenia (SCZ), bipolar disorder (BIP), depression (DEP), and attention‐deficit hyperactivity disorder (ADHD) (Høegh et al., 2020; Patel et al., 2015; Skirrow, McLoughlin, Kuntsi, & Asherson, 2009; Thompson, Berenbaum, & Bredemeier, 2011) Furthermore, MOOD is a predictor and trait‐marker for both DEP and BIP (Angst, Gamma, & Endrass, 2003; Bonsall, Wallace‐Hadrill, Geddes, Goodwin, & Holmes, 2012; Henry et al., 2008; Thompson et al., 2011), and is associated with suicidality and poor treatment outcomes in multiple disorders (Marwaha, Parsons, & Broome, 2013; Patel et al., 2015).

There is mounting evidence supporting a prominent neurobiological basis to MOOD. First, twin studies have estimated 25 and 40% heritability for affect intensity and affective lability respectively, central components of MOOD (Coccaro, Ong, Seroczynski, & Bergeman, 2012). Second, symptoms mirroring MOOD can be caused by seizure activity or localized brain lesions, typically involving the prefrontal cortex, the temporal lobe, and the diencephalon (Price, Goetz, & Lovell, 2011). Third, neuroimaging, behavioral, cognitive, and electrophysiological studies have reported an array of neurobiological correlates, of which alterations in amygdala activation and connectivity between the ventromedial prefrontal cortex, amygdala, and anterior cingulate cortex are the most convincing (Broome, He, Iftikhar, Eyden, & Marwaha, 2015). In combination with its clinical significance, MOOD therefore represents a promising transdiagnostic therapeutic target that could be leveraged to develop novel treatments and inform personalized psychiatric treatment, consistent with the Research Domain Criteria framework (Harrison, Geddes, & Tunbridge, 2018). Despite this, questions remain over MOOD's neurobiological and phenomenological consistency across and within diagnostic groups, particularly in disorders such as SCZ, which is classically associated with reduced affective expression despite increased MOOD (Das, Calhoun, & Malhi, 2014; Koenigsberg, 2010).

An improved understanding of the shared genetic basis of MOOD and different psychiatric disorders may provide insights into these questions. Two large‐scale GWASs of MOOD in the UK Biobank have previously identified 46 genomic loci and strong positive genetic correlations with depression, but weak positive correlations with SCZ, BIP, and ADHD (Ward et al., 2017, 2019). This has implicated several genes in MOOD which are also implicated in psychiatric disorders, including *PLCL1* in SCZ, *PLCL2* in BIP, and *NEGR1* in DEP (Pardiñas et al., 2018; Stahl et al., 2019; Ward et al., 2019; Wray et al., 2018). Nonetheless, much of the genetic basis for MOOD and psychiatric disorders remains unexplained and individual loci linked to both have yet to be examined systematically (Girard, Xiong, Dion, & Rouleau, 2011; Ward et al., 2019). Furthermore, the identification of overlapping loci might help to disentangle the effects of different risk loci on the diverse phenomenology of psychiatric disorders and highlight neurobiological pathways with therapeutic potential (Harrison et al., 2018).

To this end, we applied statistical genetics tools to summary statistics from GWAS of MOOD, SCZ, BIP, DEP, and ADHD (Demontis et al., 2019; Pardiñas et al., 2018; Stahl et al., 2019; Ward et al., 2019; Wray et al., 2018). We used the bivariate causal mixture model (MiXeR) to estimate the total number of trait‐influencing variants shared between MOOD and psychiatric disorders (Frei et al., 2019). Since MiXeR quantifies total genetic overlap and is unable to identify shared genomic loci, we next employed the conjunctional false discovery rate (conjFDR) method to discover loci jointly associated with MOOD and each psychiatric disorder beyond genome‐wide significance (Smeland et al., 2019). Unlike genetic correlation, which provides an aggregate measure for the balance of variants with concordant and discordant effects on two phenotypes, MiXeR and conjFDR are able to identify genetic overlap irrespective of effect direction (Smeland, Frei, Dale, & Andreassen, 2020). These methods complement genetic correlation to provide a more comprehensive overview of the genetic relationships between phenotypes. Given MOOD's increased prevalence across multiple diagnostic categories, we also aimed to identify loci that were common to MOOD and more than one psychiatric disorder, representing “transdiagnostic” MOOD loci. Finally, the conjFDR method also leverages cross‐phenotype enrichment to boost the power to identify novel genomic loci for each phenotype, thus contributing to efforts to explain greater proportions of psychiatric disorders' SNP‐based heritability (Girard et al., 2011; Smeland et al., 2019).

## 2 METHODS

### 2.1 Samples

We acquired summary statistics from a GWAS of MOOD in the UK Biobank (*n* = 363,705) (Ward et al., 2019). MOOD was assessed by a yes/no questionnaire item “does your mood often go up and down?” (Ward et al., 2019). Individuals with a self‐reported history of DEP, BIP, SCZ, “nervous breakdown,” self‐harm, suicide attempt or psychotropic medication‐use were excluded from the original MOOD GWAS. While this measure only captures “frequent fluctuations of affect” and no other features of MOOD, for example, affect intensity (Marwaha et al., 2014), a positive response has been found to be 2.5 and 14.3 times more prevalent in people with DEP and BIP compared to controls, demonstrating its clinical relevance (Angst et al., 2003). The SCZ summary statistics comprised a meta‐analysis of CLOZUK and the Psychiatric Genomics Consortium (PGC) consisting of 40,675 cases and 64,643 controls (Pardiñas et al., 2018). The DEP summary statistics was a meta‐analysis of PGC and 23andMe, Inc. samples comprising a total of 121,198 cases and 329,421 controls (Wray et al., 2018). BIP and ADHD summary statistics were acquired from the latest PGC GWAS, comprising 41,917 cases and 371,549 controls for BIP (Mullins et al., 2021) and 19,099 cases and 34,194 controls for ADHD (Demontis et al., 2019). Since the conjFDR estimate may be inflated due to sample overlap and UK Biobank participants were included in the BIP sample (*n* cases = 1,454; *n* controls = 58,113), we performed a sensitivity analysis excluding UK Biobank participants (*n* cases = 40,463; *n* controls = 313,436). This was not required for MiXeR analysis as MiXeR is tolerant to sample overlap. All summary statistics for the main analysis comprised participants of European descent. We also included height as a nonpsychiatric comparator (*n* = 709,706) (Yengo et al., 2018). PGC East Asian SCZ sample (cases = 22,778, controls = 35,362) (Lam et al., 2019) and FinnGenn BIP (cases = 4,501, controls = 192,220) and DEP samples (cases = 17,794, controls = 156,611) (FinnGen, 2020) were used for replication (Data S1). The Norwegian Institutional Review Board: Regional Committees for Medical and Health Research Ethics (REC) South‐East Norway evaluated the current protocol and found that no additional ethical approval was required because no individual data were used. The authors assert that all procedures contributing to this work comply with the ethical standards of the relevant national and institutional committees on human experimentation and with the Helsinki Declaration of 1975, as revised in 2008.

### 2.2 Data analysis

MiXeR v1.3 was applied to MOOD and each of SCZ, BIP, DEP, ADHD, and height (Frei et al., 2019). MiXeR first uses a univariate gaussian mixture model to quantify the polygenicity of each trait from GWAS summary statistics, expressed as the number of “trait‐influencing” variants (also referred to as “causal” variants). Next, a bivariate gaussian mixture model is constructed to quantify the additive genetic effect of four components: (a) variants not influencing either phenotype; variants uniquely influencing either the (b) first or (c) second phenotype and (d) variants influencing both phenotypes. Results are visualized as a Venn diagram. MiXeR also calculates the genetic correlation between phenotypes and estimates the proportion of shared variants with concordant effect direction on both phenotypes. Estimates and standard errors are calculated by performing 20 iterations using 2 million randomly selected SNPs for each iteration, followed by random pruning at a linkage disequilibrium threshold of *r* ^2^ = 0.8. Model fit is based on likelihood maximization of signed test statistics (GWAS z‐scores), evaluated by the Akaike Information Criterion (AIC) and visualised by modeled versus observed conditional quantile‐quantile (Q‐Q) plots.

We next employed conjFDR, which has been described previously in detail (Andreassen et al., 2015; Smeland et al., 2019), to identify SNPs jointly associated with MOOD and each psychiatric disorder. Briefly, conditional Q‐Q plots were constructed to visualize cross‐trait polygenic enrichment of SNP associations between MOOD and each psychiatric disorder (Data S1). Cross‐trait enrichment was leveraged within a Bayesian statistical framework to boost the power to discover shared genetic loci beyond genome‐wide significance. Computed as the maximum of two mutual conditional FDR values (Data S1), the conjFDR value provides an estimate for the posterior probability that a SNP is not associated with either trait or both traits. SNPs with a conjFDR <0.05 were assigned statistical significance.

The consistency of genetic effects in independent samples was evaluated using an en‐masse sign concordance test (Data S1) (Lee et al., 2018; Savage et al., 2018; Trubetskoy et al., 2022).

### 2.3 Genomic loci definition

Independent genomic loci jointly associated with MOOD and each psychiatric disorder were defined using the FUMA protocol (Watanabe, Taskesen, van Bochoven, & Posthuma, 2017). Significant, independent SNPs were defined as conjFDR <0.05 and *r* ^2^ < 0.6. Lead SNPs were chosen if they were in approximate linkage equilibrium with each other (*r* ^2^ < 0.1). Transdiagnostic loci were defined as physically overlapping loci which shared at least one candidate SNP with conjFDR ≤0.05 across two or more MOOD/psychiatric disorder conjunctional analyses. Effect directions within transdiagnostic loci were evaluated by comparing effect sizes of the SNP with the lowest maximum conjFDR value within the overlapping region from each MOOD/psychiatric disorder analysis, defined as the “transdiagnostic lead SNP.” LD data was calculated using the European population of the 1,000 genomes project reference panel (Auton et al., 2015).

### 2.4 Functional annotation

Candidate SNPs, defined as any SNP within each jointly associated genomic locus with a conjFDR value <0.10 and an LD *r* ^2^ ≥ 0.6 with an independent significant SNP, were functionally annotated using FUMA using default parameters (Watanabe et al., 2017). A lower conjFDR threshold for candidate SNPs was employed to maximize the probability that putative causal SNPs are captured for functional annotation, consistent with previous primary GWAS (Lee et al., 2018) and conjFDR studies (Bahrami et al., 2021; Hindley et al., 2021). SNPs were mapped to putative causal genes using three strategies: (a) positional mapping, (b) expression quantitative trait locus (eQTL) mapping, and (c) chromatin interaction mapping (Watanabe et al., 2017). We defined a subset of “credible mapped genes” as those that were mapped by all three strategies. We conducted Gene Ontology gene‐set analyses using FUMA (Watanabe et al., 2017) on credible mapped genes for each MOOD/psychiatric disorder pair. See Data S1 for further details.

### 2.5 Data availability

All GWAS summary statistics are publicly available besides 23andMe DEP data (Data S1). The code for all analyses can be accessed at https://github.com/precimed.

## 3 RESULTS

### 3.1 Using MiXeR to estimate total polygenic overlap

Univariate MiXeR demonstrated MOOD to be highly polygenic, with 10,400 (*SD* = 400) variants predicted to influence MOOD, comparable to the complex polygenic architectures of psychiatric disorders (Table S1, Figure S1).

**FIGURE 1:**
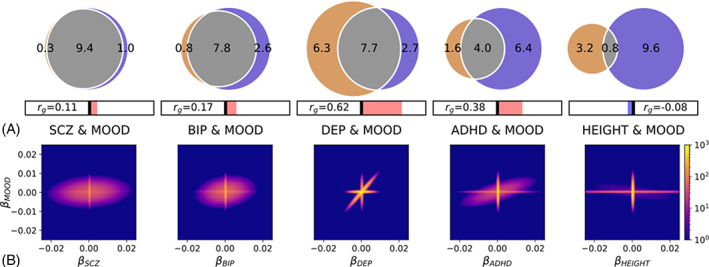
(a) Total number of shared variants between mood instability (MOOD, blue) and schizophrenia (SCZ), bipolar disorder (BIP), major depression (DEP), attention‐deficit hyperactivity disorder (ADHD), and height, as estimated by MiXeR. Venn diagrams representing the proportion of unique and shared variants associated with MOOD and each of SCZ, DEP, BIP, ADHD, and height. Polygenic overlap is represented in gray. The numbers indicate the estimated quantity of variants in thousands per component that explains 90% of SNP heritability for each phenotype. The size of the circle reflects the extent of polygenicity for each trait. Genetic correlation (*r*_g_) is represented in the horizontal bars beneath the Venn diagrams. Right of the central bar (red) indicates positive *r*_g_ and left of the central bar (blue) indicates negative *r*_g_. (b) MiXeR density plots illustrating the number of variants (color scale, blue to yellow) with a given MiXeR‐modelled effect size (*β)*, for MOOD (*y* axis) and each of SCZ, BIP, DEP, ADHD, and height (*x* axis). Extensive polygenic overlap of concordant and discordant variants is observed for MOOD and SCZ and BIP. The plots of MOOD and ADHD and DEP also illustrate extensive polygenic overlap, but most variants have concordant effects. The plot of MOOD and height indicates that most variants influencing each trait have little to no effect on the other trait.

Bivariate MiXeR analysis revealed substantial overlap between MOOD and all four disorders (Figure 1a, Table S1), both in the presence of moderate positive genetic correlation (DEP and ADHD) and minimal genetic correlation (SCZ and BIP). This occurs due to a pattern of mixed effect directions among shared variants, that is, a balance of variants with concordant and discordant effects on each trait cancel each other out resulting in minimal genetic correlation despite extensive polygenic overlap. For example, the overlap between SCZ and MOOD was particularly striking, with 9,400 (*SD* = 400) shared variants, representing 97% variants influencing SCZ and 90% variants influencing MOOD, despite weak positive genetic correlation (*r*_g_ = 0.11, *SE* = 0.0089). There was also weak positive genetic correlation (*r*_g_ = 0.10, *SE* = 0.0096) but fewer shared variants between BIP and MOOD, with 7,800 (*SD* = 600) shared variants which represented smaller proportions of trait‐influencing variants (91 and 75% for BIP and MOOD, respectively). The proportions of shared variants predicted to have concordant effects on MOOD and each of SCZ (54%, *SD* = 0.4%) and BIP (57%, *SD* = 0.5%) were consistent with extensive overlap and weak genetic correlation.

In comparison, DEP (*r*_g_ = 0.62, *SE* = 0.011) and ADHD (*r*_g_ = 0.38, *SE* = 0.012) possessed stronger positive genetic correlations with MOOD (replicating previous findings in DEP) (Ward et al., 2019). A total of 7,700 (*SD* = 300) variants were estimated to be shared between DEP and MOOD, representing 55% DEP‐influencing variants and 74% MOOD‐influencing variants. The high number of DEP‐specific variants relative to the other disorders (6,300, *SD* = 500, 45%) was likely due to DEP's extensive polygenicity (14,000, *SD* = 600) and may relate to the clinical heterogeneity of the disorder (Frei et al., 2019; Holland et al., 2020). ADHD was found to share 4,000 (*SD* = 600) variants, representing 71% ADHD influencing variants and 38% MOOD influencing variants. The high proportion of MOOD‐specific variants (6,400, *SD* = 600, 62%) is related to ADHD's lower polygenicity (5,600, *SD* = 400) relative to MOOD. Consistent with the stronger positive genetic correlations, there were higher proportions of shared variants predicted to have concordant effects, with 94% (*SD* = 2.8%) concordant for DEP and MOOD and 77% (*SD* = 6%) for ADHD and MOOD. Given the extensive polygenic overlap across phenotypes, we applied the MiXeR model to height and MOOD as a nonpsychiatric comparator. MiXeR estimated 800 shared variants (*SD* = 200) and minimal negative correlation (*r*_g_ = −0.08, *SE* = 0.0083). AIC and conditional QQ plots to assess MiXeR model fit are described in Data S1 and Figure S2.

The relationship between the number of shared variants and genetic correlation is illustrated in density plots in Figure 1b, in which the effect of each variant on MOOD is plotted against its effect on each psychiatric disorder and height. For SCZ and BIP, a large proportion of variants effect both phenotypes (oval) but these are distributed evenly between regions indicating concordant effects (top‐right and bottom‐left quadrants) and discordant effects (top‐left and bottom‐right quadrants). The effects of overlapping SNPs cancel each other out leading to weak genetic correlation despite substantial overlap. For DEP and ADHD, most variants have concordant directions, illustrated by the preponderance of variants in the top‐right and bottom‐left quadrants. This results in polygenic overlap and stronger positive genetic correlations. The MOOD and height subplot reveals that most variants affecting one trait do not influence the other and vice versa. Almost all associated variants are therefore plotted close to *β* = 0 for one or the other phenotype (horizontal and vertical lines), indicating minimal genetic overlap and weak genetic correlation.

### 3.2 Identifying genomic loci shared between MOOD and psychiatric disorders

We computed conjFDR values for each SNP present in both primary GWASs. The conjFDR value is defined as a conservative estimate for the probability that a given SNP is not associated with either phenotype. In line with previous publications, we set a threshold of conjFDR <0.05 to identify SNPs with evidence of a joint association with both phenotypes (Smeland et al., 2019). By leveraging cross‐trait enrichment and employing a Bayesian statistical framework (Figures S3–S4), conjFDR identifies jointly associated loci beyond genome‐wide significance.

At conjFDR <0.05, MOOD was jointly associated with SCZ at 102 independent genomic loci, BIP at 60 loci, DEP at 163 loci, and ADHD at 28 loci (Tables 1, S2). Among these, 246 were novel in MOOD, 26 in SCZ, 22 in BIP, 92 in DEP, and 12 in ADHD, demonstrating conjFDR's ability to boost the power to discover novel loci. On comparing the effect direction of jointly associated lead SNPs, 58.9% (60/102) were concordant for SCZ and MOOD, 65.0%% (39/60) for BIP and MOOD, 96.3% (157/163) for DEP and MOOD, and 96% (27/28) for ADHD and MOOD. These figures closely resemble the MiXeR estimates for MOOD and SCZ (54%), BIP (57%), and DEP (94%) but are somewhat discrepant from the estimate for MOOD and ADHD (77%). This is likely due to the small number of loci identified in this analysis. Functional annotation analyses for individual analyses are presented in Data S1 and Tables S3–S6. Our sensitivity analysis excluding UK Biobank participants from the BIP sample identified 55 shared genetic loci, all of which were identified by our main analysis, indicating minimal effect of sample overlap on our results (Table S4).

**TABLE 1:** Summary of conjunctional FDR (conjFDR) results for mood instability (MOOD) and each of schizophrenia (SCZ), bipolar disorder (BIP), major depression (DEP), and attention‐deficit hyperactivity disorder (ADHD). Number of joint loci at genome‐wide significance (*p* < 5 x 10^−8^), number of joint loci identified at conjFDR <0.05, number of novel loci in each phenotype, number of loci with concordant effect directions in discovery and replication samples, and number of transdiagnostic loci (overlapping between two or more Psych) are presented for each analysis. Psych = psychiatric disorders. There was no replication cohort available for ADHD. The sample sizes of the original Psych GWAS and the number of overlapping loci at genome‐wide significance in the original GWAS (5 × *p* < 10^−8^) are provided for comparison.

Psych	Psych GWAS (*n*)	Joint loci with MOOD at *p* < 5 × 10^−8^ (n)	Joint loci with MOOD at conjFDR <0.05 (n)	Novel loci in MOOD (*n*)	Novel loci in psych (*n*)	Joint loci with concordant lead SNPs in replication cohort (*n*)	Transdiagnostic loci (*n*)
SCZ	105,318	40	102	71	26	74	41
BIP	413,463	5	60	42	22	39	35
DEP	450,619	29	163	140	92	121	38
ADHD	53,293	2	28	17	12	N/A	11

### 3.3 Consistency of genetic effects in independent samples

When comparing the effect directions of lead SNPs in discovery and replication samples, there was significant en masse sign concordance for SCZ (74/96 concordant; *p* = 4.72e^−8^), BIP (39/56 concordant; *p* = 0.0023), and DEP (121/154 concordant; *p* = 2.63e^−13^). The discrepancy in the number of lead SNPs was due to missing lead SNPs in replication samples (SCZ = 6; BIP = 4; DEP = 9). We did not have access to sufficiently large independent datasets for MOOD or ADHD.

### 3.4 Identifying and characterizing transdiagnostic loci

**FIGURE 2:**
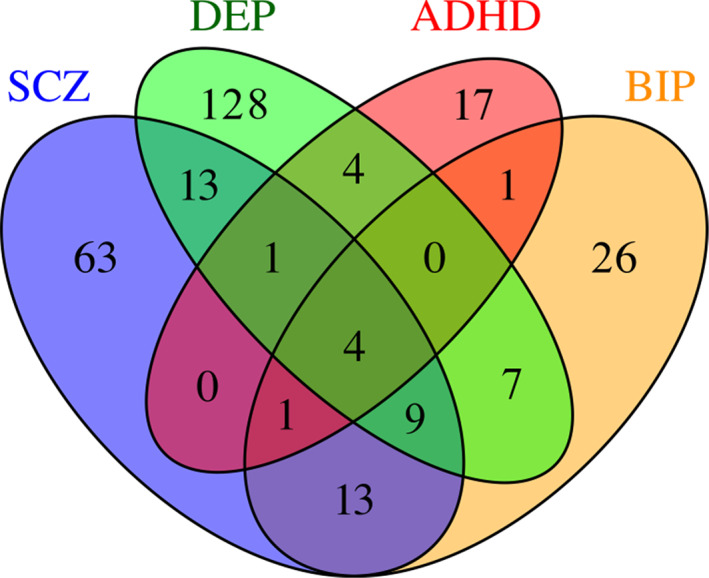
The distribution of transdiagnostic mood instability loci. Venn diagram showing the numbers of diagnosis‐specific and transdiagnostic loci across each MOOD and psychiatric disorder conjFDR analysis. ADHD, attention‐deficit hyperactivity disorder; BIP, bipolar disorder; DEP, major depression; SCZ, schizophrenia

A total of 53 loci were associated with MOOD and two or more psychiatric disorders (Table S7). Among these, 38 were associated with MOOD and two disorders, 11 with MOOD and three disorders, and 4 with MOOD and all four disorders. Seven transdiagnostic loci had divergent effect directions on psychiatric disorders, but BIP was always concordant with SCZ (*n* = 27) and DEP was always concordant with ADHD (*n* = 18). The distribution of these loci is summarized in Figure 2. Loci overlapping across three or more psychiatric disorders are presented in Table 2.

**TABLE 2:** Transdiagnostic loci jointly associated with mood instability (MOOD) and psychiatric disorders (Psych) across three or more disorders

Chr	Psych	Min‐max BPs	Trans‐diagnostic lead SNP	conjFDR	Concordant effects	Novel in psych	Novel in MOOD	Mapped genes
2	SCZ	22,430,795–22,545,027	rs13387284	0.029	TRUE	x	TRUE	*AC068490.2* ^a^
	BIP	22,430,795–22,606,275		0.035	TRUE	x		
	DEP	22,430,795–22,606,275		0.010	TRUE	x		
				0.029	TRUE	x		
	ADHD	22,430,795–22,493,637						
2	SCZ	57,942,987–58,505,679	rs2717039	0.029	TRUE	x	x	***VRK2**, FANCL*, *BCL11A*
				0.032	TRUE	x		
	BIP	57,956,088–58,444,610		0.010	TRUE	x		
	DEP	57,942,987–58,484,172						
4	SCZ	122,913,532–123,558,330	rs10014468	0.019	x	x	x	*TRPC3*, *KIAA1109*, *IL21*
	BIP	123,026,869–123,558,330		0.0048	x	x		
	DEP	123,052,343–123,558,330		0.0015	TRUE	x		
5	SCZ	103,791,044–104,055,261	rs2447832	0.044	TRUE	x	x	*RP11‐6N13* ^a^, *CTD‐2374C24.1* ^a^
	BIP	103,671,867–104,082,179		0.036	TRUE	x		
				0.013	TRUE	x		
	DEP	103,671,867–104,082,179						
				0.033	TRUE	x		
	ADHD	103,671,867–104,082,179						
7	SCZ	1,873,756–2,110,850	rs55790766	0.026	x	x	x	***AC110781.3***, *INTS1*, *MAFK*, *TMEM184A*, *PSMG3*, *ELFN1*, *MAD1L1*, *FTSJ2*, *NUDT1*
	BIP	1,882,795–2,110,850		0.011	x	x		
	DEP	1,860,733–2,247,403		0.005	TRUE	x		
	ADHD	1,873,756–2,110,850		0.037	TRUE	x		
7	SCZ	82,386,297–82,641,937	rs2158220	0.006	TRUE	x	TRUE	*HGF*, *PCLO*
	BIP	82,376,952–82,555,669		0.01	TRUE	x		
	DEP	82,386,297–82,557,937		0.012	TRUE	x		
8	SCZ	8,088,230–11,417,790	rs2952245	0.028	x	x	x	***MRSA*** ^c^
	BIP	7,632,319–11,830,150		0.044	x	x		
	DEP	10,121,605–10,435,915		0.036	TRUE	x		
10	SCZ	106,453,832–106,640,653	rs2496014	0.016	TRUE	x	x	*SORCS3*
	BIP	106,455,520–106,640,653						
	DEP	106,405,854–106,830,537		0.022	TRUE	x		
				0.006	TRUE	x		
	ADHD	106,392,549–106,640,653						
				0.016	TRUE	x		
11	SCZ	113,185,591–113,692,660	rs2514218	7.37e^−7^	TRUE	x	x	***TTC12***, ***DRD2***, *AP002884.3*, *BCO2*, *PLET1*, *AP002884.2*, *TMPRSS5*, *ZBTB16*
	BIP	113,241,877–113,451,229		0.001	TRUE	x		
	DEP	113,166,310–113,692,660		0.0015	TRUE	x		
12	SCZ	2,474,661–2,523,772	rs2239063	0.038	TRUE	x	TRUE	*CACNA1C*
	BIP	2,474,661–2,523,772		0.043	TRUE	x		
	DEP	2,465,364–2,523,772		0.016	TRUE	x		
18^b^	SCZ	50,517,509–51,055,069	rs1367635	0.0032	TRUE	x	x	*DCC*
	BIP	50,711,776–50,907,127		0.034	TRUE	x		
	DEP	50,358,109–51,055,069		0.0035	TRUE	x		
18^b^	SCZ	50,517,509–51,055,069	rs7506904	0.049	TRUE	x	x	*DCC*
	DEP	50,197,439–51,055,069		0.048	TRUE	x		
	ADHD	50,358,109–51,055,069		0.046	TRUE	x		
18	SCZ	52,720,948–53,474,904	rs4505420	0.0032	TRUE	x	x	*RAB27B*, *TXNL1*, *WDR7*
	BIP	52,720,948–52,827,668		0.0083	TRUE	TRUE		
	DEP	52,520,149–53,424,880		0.0098	TRUE	x		
20	SCZ	44,680,853–44,749,251	rs6032660	0.044	TRUE	TRUE	x	***SLC12A5***, ***CD40***, *UBE2C*, *ZSWIM1*, *SPATA25*, *NEURL2*, *CTSA*, *PCIF1*, *AL162458.1*, *NCOA5*, *ELMO2*
	BIP	44,680,412–44,747,947		0.047	TRUE	x		
	ADHD	44,680,853–44,749,251		0.042	TRUE	x		
22	SCZ	41,080,566–42,248,289	rs80533	0.01	TRUE	x	x	***MCHR1***, ***SLC25A17***, ***XPNPEP3***, ***RBX1***, ***EP300***, ***L3MBTL2***, ***RANGAP1***, ***ZC3H7B***, *SGSM3*, *TOB2*, *PHF5A*, *ACO2*, *POLR3H*, *MEI1*, *WBP2NL*
	BIP	41,080,566–41,404,511		0.021	TRUE	x		
	DEP	41,080,566–42,216,326		0.0067	TRUE	x		

*Note*: Minimum and maximum base pairs (BPs), “transdiagnostic lead SNPs”, and conjunctional false discovery statistics (conjFDR) are presented for each locus. The concordance of effect direction and novelty of a locus for MOOD and each Psych is indicated by “TRUE.” Protein‐coding genes mapped to candidate SNPs from each MOOD/Psych analysis are presented. Genes mapped by all three mapping strategies (credible genes) are in bold.

^a^ If there were no protein‐coding genes mapped to a locus, nonprotein‐coding‐mapped genes are presented.

^b^ Loci are physically overlapping but there was no candidate SNP with conjFDR <0.05 across all four analyses.

^c^ Locus spans 8p23 inversion region with complex linkage disequilibrium. This biases gene‐mapping strategies so only a single mapped gene is presented.

**FIGURE 3:**
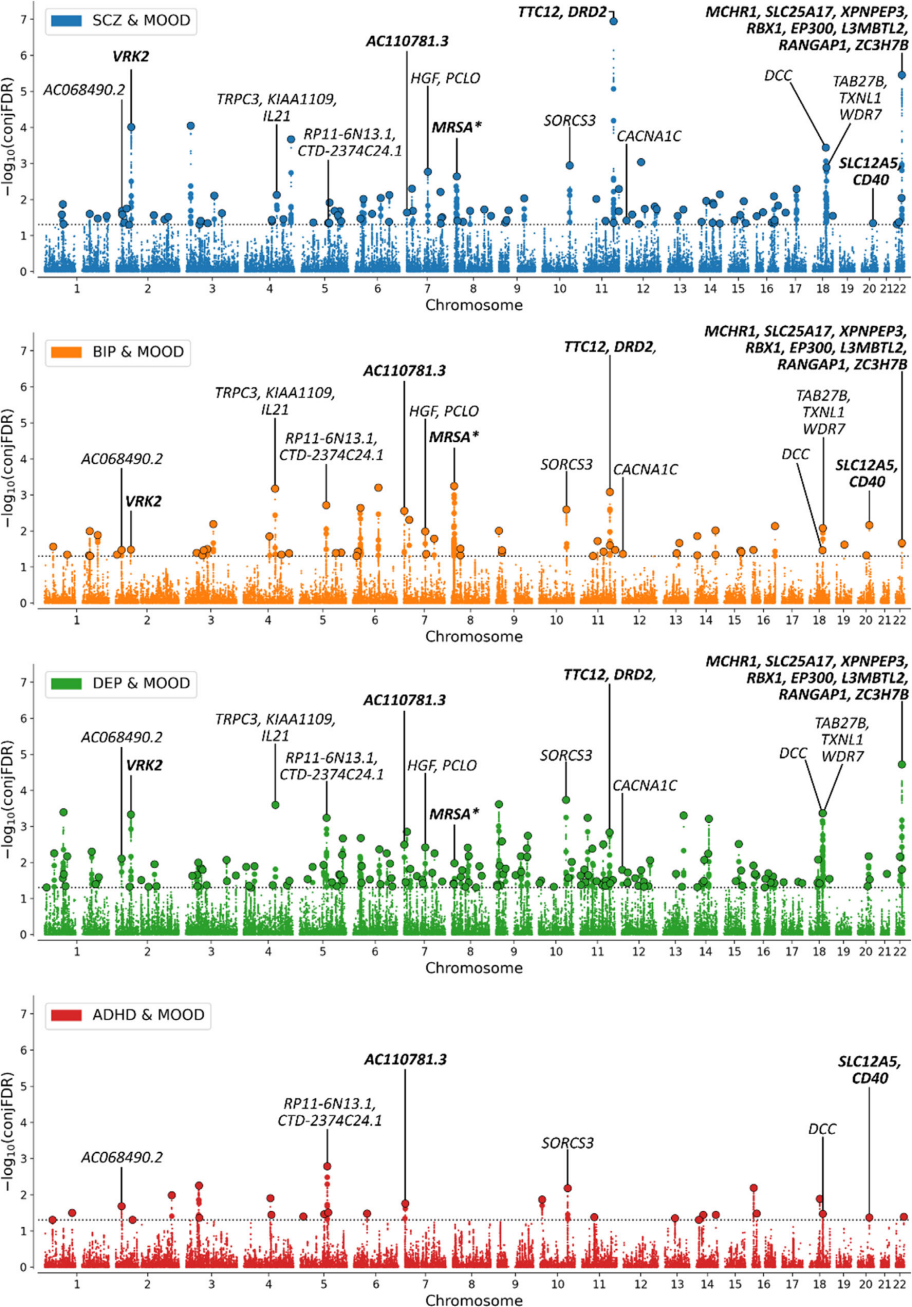
Manhattan plot showing ‐log10 transformed conjunctional FDR (conjFDR) values (*y* axis) for mood instability (MOOD) and (a) schizophrenia (SCZ, blue), (b) bipolar disorder (BIP, orange), (c) major depression (DEP, green), and (d) attention‐deficit hyperactivity disorder (ADHD, red) against chromosomal position (*x* axis) for each SNP. The dotted line represents conjFDR <0.05 significance threshold. Black circles represent lead SNPs. Lead SNPs from transdiagnostic loci across three or more disorders are annotated with mapped genes. N.B. Not all mapped genes for each locus are presented due to space limitations. Credible genes (bold) were prioritized followed by protein‐coding genes and then nonprotein‐coding genes. *Locus spans 8p23 inversion region with complex linkage disequilibrium. This biases gene‐mapping strategies so only a single mapped gene is presented.

We next identified 1,179 genes that were mapped to candidate SNPs from two or more psychiatric disorder/MOOD pairs, 64 of which were mapped by all three strategies (Table S8). Figure 3 illustrates the chromosomal distribution of the shared loci for each phenotypic pairing alongside mapped genes for each transdiagnostic locus overlapping across three or more disorders. Among these, *VRK2*, *KIAA1109*, *AC110781.3*, *PCLO*, *TMPRSS5*, and *EP300* were all mapped to nonsynonymous exonic SNPs. Furthermore, *VRK2*, *AC110781.3*, and *EP300* were mapped by all three mapping strategies, including eQTLs in the substantia nigra (*VRK2*), caudate, hypothalamus and nucleus accumbens (*AC110781.3*), and the cerebellum and hypothalamus (*EP300*). *VRK2* is a serine threonine kinase which has previously been implicated in SCZ, BIP and DEP and plays a role in neuronal proliferation and migration (Li & Yue, 2018). *AC110781.3* is a gene of unknown function expressed within 13 different brain tissues, with greatest expression in the cortex, amygdala, and hippocampus (Figure S5). It was also mapped to a locus associated with all four disorders, but with opposite effect directions on SCZ and BIP vs. DEP and ADHD. Finally, *EP300* is a histone acetyltransferase implicated in cell proliferation and differentiation. Other notable genes mapped to transdiagnostic loci include the dopamine receptor D2 gene (*DRD2*), the calcium channel voltage‐gated channel subunit *CACNA1C*, and the neuron‐specific potassium/chloride transporter *SLC12A5*.

A single gene‐set, “Synapse organization” was significantly enriched with mapped genes from all four analyses. There were a further six gene‐sets enriched with mapped genes for MOOD and each of SCZ, BIP, and DEP, although there was extensive overlap in genes across the different gene‐sets. All gene‐sets besides “Neuron part” were either directly or indirectly related to synaptic structure (“Synapse,” “Synapse part,” “Postsynapse,” “Synaptic membrane,” “cell projection part”) (Table S9). Interestingly, when linking mapped genes from each gene‐set back to their associated genomic locus, there was a divergent pattern of effect directions with SCZ (51.4–61.8%) and BIP (58.3–70.6%) showing a pattern of mixed effect directions with MOOD, while DEP (97.4–100%) and ADHD (100%) were almost entirely concordant. This is consistent with the patterns of effect directions estimated by MiXeR and observed in jointly associated loci identified by conjFDR (Table S10). This indicates that the divergent pattern of effect directions persists at the level of specific gene‐sets.

## 4 DISCUSSION

In this combined GWAS analysis, we used MiXeR to reveal extensive polygenic overlap between MOOD and each of SCZ, BIP, DEP, and ADHD despite divergent patterns of genetic correlations. A large proportion of the genetic variants linked to psychiatric disorders also influence MOOD, but the number of shared and trait specific variants and the balance of protective and risk‐enhancing variants differ across diagnostic groups. Using conjFDR, MOOD was jointly associated with SCZ at 102 loci, BIP at 60 loci, DEP at 163 loci, and ADHD at 28 loci, representing 259 unique genomic loci jointly associated with MOOD and psychiatric disorders. Of these, 220 were novel in MOOD and 152 were novel in psychiatric disorders. Replication analysis provided evidence of consistent genetic effects in independent SCZ, BIP, and DEP samples. We identified 53 transdiagnostic loci that were overlapping across MOOD and two or more psychiatric disorders, implicating 1,179 putative transdiagnostic genes with an apparent convergence on synaptic gene‐sets, although a divergent pattern of effect directions persisted within shared gene‐sets. These findings have implications for how the genetic risk of mental‐health‐related traits is conceptualized and suggests differences in the neurobiological basis of MOOD across different psychiatric disorders, including the possibility of genetically influenced subgroups of patients with more or less prominent MOOD. We also highlight genes that are likely to influence MOOD across several diagnoses, indicating high relevance for future in vitro and in vivo investigation.

First, 55–97% of disorder associated variants were predicted to influence MOOD, raising questions about the specificity of the genetic architecture of these complex polygenic psychiatric disorders and related traits. Our findings compliment evidence that a large proportion of genetic variants are not unique for a given mental trait or disorder (Smeland et al., 2020; Smeland, Frei, Dale, & Andreassen, 2020), but influence multiple mental phenotypes to different degrees. As such, the distinct SNP‐based risk profiles for different mental health‐related traits are not merely defined by unique nonoverlapping sets of genetic variants, but largely accounted for by a set of nonspecific pleiotropic genetic variants showing different strengths of association and effect across these phenotypes (Smeland, Frei, Dale, & Andreassen, 2020). Although this hypothesis warrants further interrogation, it suggests that novel approaches are needed to account for the substantial pleiotropy we predict in order to robustly distinguish the genetic risk for different mental traits and disorders (Smeland et al., 2020). Furthermore, this places emphasis on identifying disease specific variants that may disproportionately affect the development of a specific phenotype, individually and/or collectively, to inform precision medicine approaches in psychiatry.

Secondly, MOOD has gained interest due to its prevalence across diagnoses and its prominent neurobiological basis (Broome et al., 2015), implying that it may represent a novel treatment target (Broome et al., 2015; Koenigsberg, 2010). To some extent, this is supported by the large degree of shared genomic loci and corresponding mapped genes identified. However, there were differences in genetic correlations and effect directions of shared loci, with stronger positive correlations and higher proportions of loci with concordant effects in DEP and ADHD compared to weak correlations and lower proportions of loci with concordant effects in SCZ and BIP. This pattern persisted within specific gene‐sets identified across multiple analyses. This implies that there may be mechanistic differences in MOOD across the four psychiatric disorders. It is important to note that this measure only reflects one aspect of MOOD, which may explain the lack of correlation with SCZ and BIP, particularly given MOOD's strong clinical association with BIP. Nonetheless, it is tempting to speculate that MOOD experienced in DEP and ADHD has a similar neurobiological relationship whereas MOOD in BIP and SCZ may reflect a different underlying etiological mechanism. This is relevant as such differences may limit the potential for transdiagnostic pharmacological interventions. Alternatively, the current findings are also consistent with subgroups characterized by higher or lower MOOD within diagnostic categories, in line with clinical observations (Ducasse et al., 2017). Above all, these findings emphasize the importance of exploring the neurobiological and phenomenological differences in MOOD across diagnostic groups.

To characterize MOOD's neurobiological underpinnings, we used three gene‐mapping strategies to identify credible mapped genes for all jointly associated loci. Among these, *AC110781*.*3* was mapped to a nonsynonymous exonic SNP jointly associated with MOOD and all four psychiatric disorders. *AC110781*.*3* is a protein‐coding gene of unknown function that is expressed in the cortex, amygdala, and hippocampus. In addition to previously being implicated in schizophrenia (Huckins et al., 2019) and risk‐taking behavior (Karlsson Linnér et al., 2019), we also recently linked *AC110781*.*3* to multiple sleep phenotypes and BIP, DEP, and SCZ (O'Connell et al., 2021). This suggests *AC110781*.*3* influences multiple diverse phenotypes and may represent a promising candidate for further *in vitro* and *in vivo* investigation. We also identified several well‐established psychiatric risk genes, including *VRK2* (Li & Yue, 2018), *CACNA1C* (Moon, Haan, Wilkinson, Thomas, & Hall, 2018), and *DRD2* (Schizophrenia Working Group of the Psychiatric Genomics Consortium, 2014), although an association with mood instability has not previously been described in the literature. The convergence of transdiagnostic‐mapped genes on synaptic structure builds on Ward et al.'s finding in the primary MOOD GWAS that mapped genes were associated with synaptic transmission.

Finally, while mood instability has a prominent genetic component, it remains influenced by environmental factors (Coccaro et al., 2012; Ward et al., 2019). Future work focusing on gene‐environmental interplay, particularly in relation to childhood trauma which has been found to correlate with the development of mood instability (Marwaha et al., 2016), would be of high interest.

There were limitations to the current study. First, due to available sample sizes and multiancestral differences in allele frequency and LD structure, we were unable to include multiancestral samples. In particularly, the use of multiancestry samples for conjFDR analysis may result in false positive associations, and there are currently no non‐European MOOD samples of sufficient size to perform a non‐European conjFDR analysis with MOOD. Furthermore, people from higher socioeconomic classes and with better general health are over‐represented within the UKB, and so these findings may not be generalizable to the wider population. Nonetheless, we used an East‐Asian SCZ sample for replication to show consistent genetic effects of our discovered loci in a non‐European sample. More representative population samples, such as the All‐of‐Us study, will enable more representative genetic research in the coming years (Mapes et al., 2020). Second, due to the small sample size of the most recent borderline personality disorder GWAS (*n* = 2,579), we were unable to include it in the current analysis despite its primacy in MOOD (Koenigsberg, 2010). This analysis should be repeated as sample sizes increase to include other relevant disorders, identify more transdiagnostic loci, and validate MiXeR's predictions. Third, the measure of MOOD was based on a single, binary questionnaire item that did not measure affect intensity, regulation of affect or behavioral sequelae, and did not specify the timeframe (Marwaha et al., 2014). This may have contributed to the lack of genetic correlations with SCZ and BIP. Nonetheless, a simple measure was necessary to achieve a large enough sample size for gen. A previous GWAS using more complete measures had a substantially smaller sample size and failed to identify genome‐wide significant loci (Gisbert et al., 2019). Moreover, the same binary questionnaire item is associated with BIP and DEP, demonstrating its clinical relevance (Angst et al., 2003). Future work with more refined measures is required to understand how these findings relate to other dimensions of MOOD. Fourth, differences in sample size affect conjFDR's power to discover shared loci. This precludes cross‐analysis comparisons of the number of loci discovered by conjFDR. The disparity between the number of shared loci discovered and the number of shared variants predicted by MiXeR also indicates that we cannot confidently identify loci “unique” to each mental disorder, since it is possible that the lack of association is due to type II error. This will only be addressed once larger proportions of disorder‐influencing variants have been discovered. Finally, we used a hypergeometric test‐based gene set enrichment analysis. This approach is limited by the fact that it does not control for gene‐size and may be vulnerable to gene clusters. We were unable to use alternative gene‐set analyses which control for these biases, such as MAGMA (de Leeuw, Mooij, Heskes, & Posthuma, 2015), since these methods assume a uniform distribution of association *p*‐values among null SNPs which is not the case for conjFDR statistics. We attempted to alleviate the effects of these biases by using credible genes as the input list of genes which are less likely to contain multiple mapped genes from the same gene cluster. Our definition of credible genes also demanded physical proximity between SNP and gene. This excludes credible relationships between SNPs and distal genes, reducing the sensitivity of the gene‐mapping strategy. Nonetheless, eQTL and chromatin interaction mapping are sensitive to the effects of linkage disequilibrium and so are liable to generate false positives. Since we identified a large number of loci, we prioritized specificity over sensitivity to ensure robust gene‐set analyses.

In conclusion, we have discovered extensive polygenic overlap between MOOD and psychiatric disorders with divergent patterns of genetic correlation and effect directions. These results support the notion that there are common molecular pathways implicated in MOOD across diagnostic categories, but disorder specific effect size distributions indicate potential differences in MOOD's neurobiological underpinnings across diagnoses.

## AUTHOR CONTRIBUTIONS

Conceived and designed the analysis: G.H., K.S.O., O.A.A.; Contributed data or analysis tools: K.S.O., Z.R., O.F., A.M.D., O.A.A.; Performed the analysis: G.H., K.S.O., Z.R., O.F.; Drafting the article: G.H.; critical revision of the article: G.H., K.S.O., Z.R., O.F., S.B., A.S., M.C.H., W.C., N.K., A.L., L.R., C.C.F., S.D., T.V.L., A.M.D., O.B.S., O.A.A.; Final approval of the version to be published: G.H., K.S.O., Z.R., O.F., S.B., A.S., M.C.H., W.C., N.K., A.L., L.R., C.C.F., S.D., T.V.L., A.M.D., O.B.S., O.A.A.

## FUNDING INFORMATION

The authors gratefully acknowledge support from the American National Institutes of Health (EB000790, 1R01MH124839), the Research Council of Norway (RCN 229129, 213837, 223273), the South‐East Norway Regional Health Authority (2017‐112), KG Jebsen Stiftelsen (SKGJ‐MED‐008), and the PGC US Norway Collaboration (RCN# 248980). This project has received funding from the European Union's Horizon 2020 research and innovation program under grant agreement No 847776.

## CONFLICT OF INTEREST

O.A.A. has received speaker's honorarium from Lundbeck and is a consultant for Healthlytix. A.M.D. is a founder of and holds equity interest in CorTechs Labs and serves on its scientific advisory board. He is also a member of the Scientific Advisory Board of Healthlytix and receives research funding from General Electric Healthcare (GEHC). The terms of these arrangements have been reviewed and approved by the University of California, San Diego in accordance with its conflict‐of‐interest policies. Remaining authors have nothing to disclose.

## Supporting information


**Data S1** Supporting information

**Data S2** Supplementary tables

## Data Availability

All PGC data are available at https://www.med.unc.edu/pgc/download-results/, SCZ meta‐analysis of PGC and CLOZUK datasets at https://walters.psycm.cf.ac.uk/, height at https://portals.broadinstitute.org/collaboration/giant/index.php/GIANT_consortium_data_files, and FinnGen at https://www.finngen.fi/en/access_results. Full GWAS summary statistics for the 23andMe DEP dataset will be made available through 23andMe to qualified researchers under an agreement with 23andMe that protects the privacy of the 23andMe participants. Interested investigators should email dataset‐request@23andme.com and reference this paper for more information. Summary statistics for MOOD are available upon request by contacting the corresponding author of the original MOOD GWAS.
